# Mesencephalic Astrocyte-Derived Neurotrophic Factor (MANF) Elevates Stimulus-Evoked Release of Dopamine in Freely-Moving Rats

**DOI:** 10.1007/s12035-018-0872-8

**Published:** 2018-01-18

**Authors:** Juho-Matti Renko, Susanne Bäck, Merja H. Voutilainen, T. Petteri Piepponen, Ilkka Reenilä, Mart Saarma, Raimo K. Tuominen

**Affiliations:** 1 Division of Pharmacology and Pharmacotherapy, University of Helsinki, Viikinkaari 5E, P.O. Box 56, 00014 Helsinki, Finland; 2 Institute of Biotechnology, Research Program in Developmental Biology, University of Helsinki, Viikinkaari 5D, P.O. Box 56, 00014 Helsinki, Finland

**Keywords:** Glial cell line-derived neurotrophic factor (GDNF), Cerebral dopamine neurotrophic factor (CDNF), Mesencephalic astrocyte-derived neurotrophic factor (MANF), Dopamine microdialysis, Tyrosine hydroxylase (TH), Catechol-*O*-methyltransferase (COMT)

## Abstract

Neurotrophic factors (NTFs) hold potential as disease-modifying therapies for neurodegenerative disorders like Parkinson’s disease. Glial cell line-derived neurotrophic factor (GDNF), cerebral dopamine neurotrophic factor (CDNF), and mesencephalic astrocyte-derived neurotrophic factor (MANF) have shown neuroprotective and restorative effects on nigral dopaminergic neurons in various animal models of Parkinson’s disease. To date, however, their effects on brain neurochemistry have not been compared using in vivo microdialysis. We measured extracellular concentration of dopamine and activity of dopamine neurochemistry-regulating enzymes in the nigrostriatal system of rat brain. NTFs were unilaterally injected into the striatum of intact Wistar rats. Brain microdialysis experiments were performed 1 and 3 weeks later in freely-moving animals. One week after the treatment, we observed enhanced stimulus-evoked release of dopamine in the striatum of MANF-treated rats, but not in rats treated with GDNF or CDNF. MANF also increased dopamine turnover. Although GDNF did not affect the extracellular level of dopamine, we found significantly elevated tyrosine hydroxylase (TH) and catechol-*O*-methyltransferase (COMT) activity and decreased monoamine oxidase A (MAO-A) activity in striatal tissue samples 1 week after GDNF injection. The results show that GDNF, CDNF, and MANF have divergent effects on dopaminergic neurotransmission, as well as on dopamine synthetizing and metabolizing enzymes. Although the cellular mechanisms remain to be clarified, knowing the biological effects of exogenously administrated NTFs in intact brain is an important step towards developing novel neurotrophic treatments for degenerative brain diseases.

**Electronic supplementary material:**

The online version of this article (10.1007/s12035-018-0872-8) contains supplementary material, which is available to authorized users.

## Introduction

Parkinson’s disease (PD) is a progressive neurodegenerative disorder which holds an unmet need for a curative treatment. It is the most common degenerative brain disease after Alzheimer’s disease affecting approximately 2–3% of the population over 65 years of age [1]. The essential neuropathological hallmarks of PD are death of dopaminergic cell bodies in the midbrain substantia nigra pars compacta and presence of intraneuronal cytoplasmic aggregates containing misfolded α-synuclein called Lewy bodies and Lewy neurites [2, 3]. The characteristic parkinsonian motor symptoms, including bradykinesia, rigidity, resting tremor, and postural impairment, result from the degeneration of the nigrostriatal dopaminergic neurons and resultant dopamine deficiency within the dorsal striatum.

Neurotrophic factors (NTFs) are endogenous secretory proteins which promote differentiation, maintenance, function, and plasticity of the nervous system and help neurons to recover after an injury [4–7]. Due to these trophic effects, NTFs are considered as potential disease-modifying therapies for neurodegenerative disorders such as PD. Glial cell line-derived neurotrophic factor (GDNF), cerebral dopamine neurotrophic factor (CDNF), and mesencephalic astrocyte-derived neurotrophic factor (MANF) have shown neuroprotective and neurorestorative effects on lesioned dopaminergic neurons in vitro and in various animal models of PD [8–18]. In in vivo lesion models, these NTFs increase the survival of midbrain dopamine cells and fibers and improve aberrant motor performance suggesting enhanced dopaminergic function. However, if we want to look upon NTFs as a novel therapeutic approach for PD, it is crucial to understand how exogenously administered, non-physiological concentrations of NTFs influence the normal nigrostriatal neurochemistry and neurotransmitter homeostasis.

GDNF is a distant member of the TGF-β superfamily of growth factors and conveys its biological effects via receptor tyrosine kinase RET as Airaksinen and Saarma have comprehensively reviewed [5]. GDNF has been shown to potentiate the excitability of dopaminergic neurons and increase the release of dopamine in cell cultures and midbrain slices [19–22]. Intracranial administration of GDNF elevated stimulus-evoked dopamine overflow in the striatum of rats and rhesus monkeys [23–29]. However, the major limitation in the earlier in vivo studies is that they have been conducted under general anesthesia which is known to have marked effects on neurotransmission, inducing alterations in neuronal activity, neurotransmitter synthesis, release, reuptake, and metabolism [30, 31]. To the best of our knowledge, the ability of GDNF to alter dopamine release in completely non-anesthetized animals has not been studied before.

CDNF and MANF form an evolutionary conserved and structurally distinct family of NTFs [6, 12, 13]. CDNF and MANF are located intracellularly in the endoplasmic reticulum (ER), but they can be also secreted from cells [32–34]. Their mechanism of action is still unclear, although growing body of evidence suggests that CDNF and MANF play an important role in the maintenance of protein homeostasis in the ER and alleviate/regulate ER stress [35–41]. Thus far, however, the effects of intracerebrally administrated CDNF or MANF on dopaminergic neurotransmission in vivo have remained unstudied.

It has been shown that an intrastriatal injection of GDNF as well as long-term overexpression of GDNF downregulate tyrosine hydroxylase (TH), the rate-limiting enzyme in dopamine biosynthesis, in the midbrain of adult rats without affecting the total activity of the enzyme [42–44]. On the other hand, GDNF administration has been reported to increase phosphorylation of TH at Ser31 and Ser40—the serine residues controlling TH activity [28, 42, 45, 46]. To date, nothing is known about the effects of CDNF or MANF on TH activity, nor the effect of GDNF on dopamine metabolizing enzymes catechol-*O*-methyltransferase (COMT) and monoamine oxidases A and B (MAO-A and MAO-B).

The aim of the present study was to elucidate the effects of intrastriatally administrated GDNF, CDNF, and MANF proteins on dopamine release within the dorsal striatum of intact freely-moving rats. We also wanted to clarify the effect of a single intrastriatal injection of GDNF, CDNF, and MANF on in vivo activity of TH. Finally, based on the results of the microdialysis experiments, we tested the hypothesis that GDNF alters the activity of COMT, MAO-A, and MAO-B. Our results revealed divergent changes in dopamine release as well as in dopamine synthesis and metabolism after exogenously administrated NTFs. This is highly relevant information when regarding NTFs as novel therapeutic approaches for PD.

## Materials and Methods

### Experimental Animals

Male Wistar rats (RccHan:WIST, Harlan, the Netherlands), weighing 230–490 g during stereotaxic surgery, were used for all experiments. For microdialysis experiments, rats were moved from group housing (3–4 animals) to individual test cages (30 × 30 × 30 cm) after the surgery. Tap water and rat chow (Tekland Global Diet, Harlan) were available ad libitum. Rats were under a 12:12 h light-dark cycle (lights on at 6:20 am), at an ambient temperature of 20–22 °C. Stereotaxic surgeries and experiments were performed during daylight between 8:00 am and 6:00 pm. All animal experiments were in accordance with the directive of the European Parliament and of the Council on the protection of animals used for scientific purposes (Directive 2010/63/EU of the European Parliament and of the Council). Experiments were approved by the National Animal Experiment Board of Finland (ESLH-2009-05234/Ym-23 and ESAVI/198/04.10.07/2014).

### Neurotrophic Factors

Vehicle group received sterile phosphate-buffered saline (PBS). Recombinant hGDNF (2 μg/μl, reconstituted with Milli-Q® water according to the manufacturer’s instructions) was produced in *E. coli* bacterial cells (ProSpec-Tany TechnoGene Ltd., Israel). The purity of GDNF was greater than 95% as determined by SDS-PAGE electrophoresis and RP-HPLC. GDNF was tested by the manufacturer to be compliant for cell culture use in terms of lipopolysaccharide (LPS) counts in the protein sample. Recombinant hCDNF (2 μg/μl, in PBS) was produced in *Spodoptera frugiperda* (Sf9) insect cells (Biovian Oy, Finland) and recombinant hMANF (2 μg/μl, in PBS) in Chinese Hamster Ovary (CHO) mammalian cells (Icosagen AS, Estonia). Both CDNF and MANF were purified from serum-free cell supernatant using ion-exchange chromatography. The purity of CDNF and MANF was greater than 95% as determined by SDS-PAGE electrophoresis and mass-spectrometry.

### Stereotaxic Surgery

Stereotaxic surgeries were performed under isoflurane (Attane Vet 1000 mg/g, Piramal Healthcare, UK) anesthesia (3.5–4.5% during induction and 2.0–3.5% during maintenance). Rats were fixed on a stereotaxic frame (Stoelting Co., IL, USA), and the skull was exposed. Lidocaine-adrenalin solution (10 mg/ml, Orion Pharma Oyj, Finland) was used for local anesthesia and to prevent bleeding. A burr hole was made using a high-speed dental drill (Foredom SR, The Foredom Electric Co., CT, USA). A unilateral injection of GDNF, CDNF, or MANF (10 μg in 5 μl) or PBS as vehicle (5 μl) was made into the left dorsal striatum (A/P + 1.0; M/L + 2.7; D/V − 5.0 relative to the bregma, according to the rat brain atlas [47]) using an electronic microinjector (Quintessential stereotactic injector, Stoelting) and a 10-μl microsyringe (Hamilton Company, NV, USA) with a 26 gauge blunt tapered needle. The injection rate was set to 1 μl/min. At the completion of the injection, the needle was kept in place for 4 min to minimize backflow of the solution. For the microdialysis experiments, a guide cannula (BASi MD-2250, Bioanalytical Systems Inc., IN, USA) was implanted right after the NTF or vehicle injection. The tip of the cannula was lowered into the left dorsal striatum (A/P + 1.0; M/L + 2.7; D/V − 4.0 relative to the bregma, according to the rat brain atlas [47]) after which the cannula was attached to the skull with three stainless steel screws and dental cement (Aqualox, Voco Cuxhaven GmbH, Germany). To relieve postoperative pain, rats received tramadol 1 mg/kg s.c. (Tramal 50 mg/ml, Orion Pharma) at the end of the surgery, and another similar injection 12 h later if needed. After the surgery, rats were allowed to recover for 7 days before the first microdialysis experiment.

### Microdialysis

**Fig. 1:**
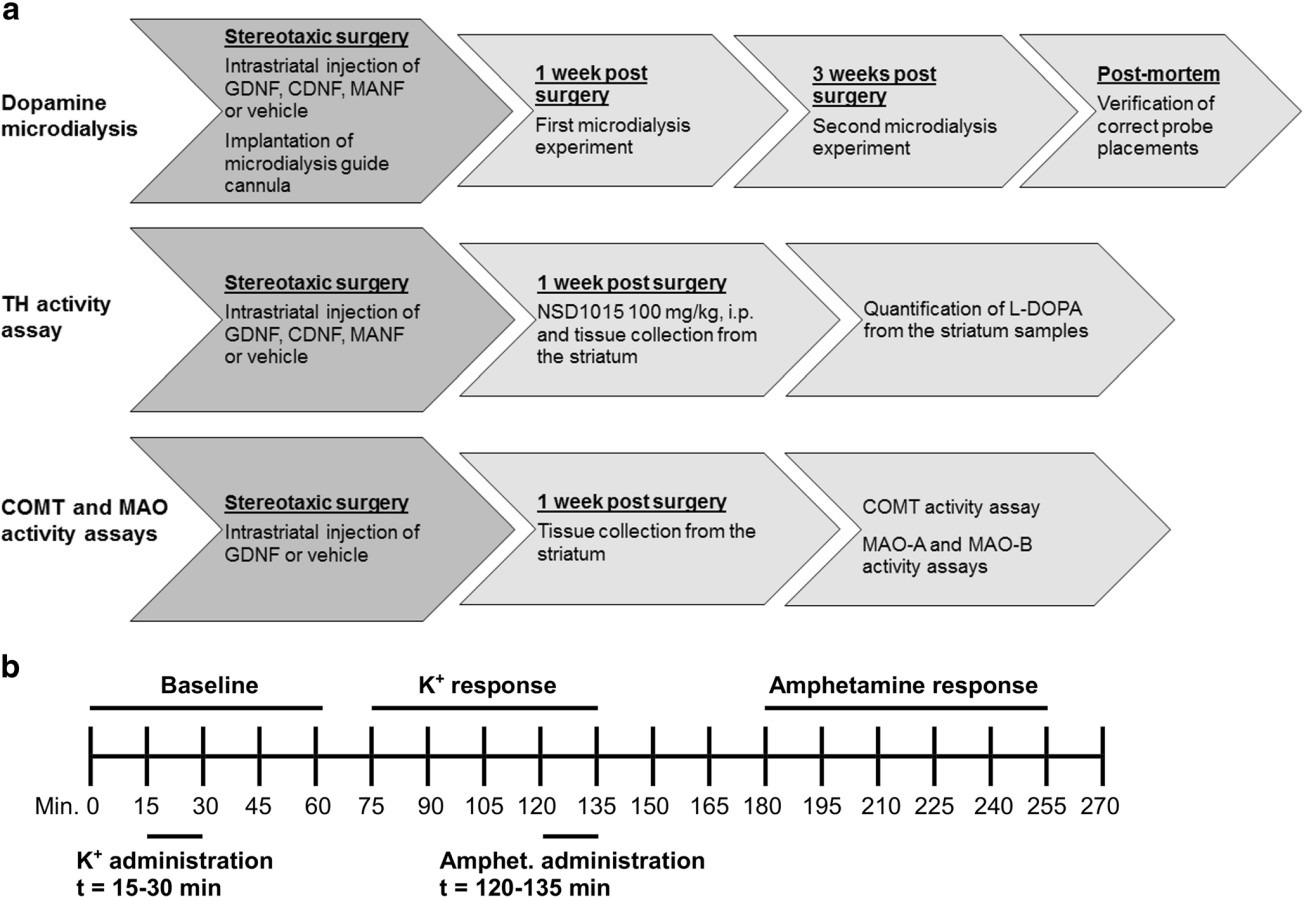
Design of the study and time course of the dopamine microdialysis experiments. **a** All rats received an intrastriatal, unilateral injection of GDNF, CDNF, MANF, or vehicle. One cohort of rats underwent dopamine microdialysis experiments 1 and 3 weeks after the NTF injection. Another cohort of rats was used to collect striatal tissue samples for in vivo TH activity measurements 1 week after the NTF treatment. NSD1015 (3-hydroxybenzylhydrazine) was administrated i.p. to inhibit aromatic amino acid dopa decarboxylase in the brain 30 min before tissue collection. From the third cohort of rats, striatal tissue samples were collected for ex vivo COMT and MAO activity measurements 1 week after the NTF treatment. **b** In the microdialysis experiments, samples were collected every 15 min for 270 min. The first four samples collected at 15, 30, 45, and 60 min were used to calculate baseline levels. Then, dopamine release was stimulated via reverse dialysis of two different stimulation solutions. First high-potassium (100 mM) perfusion solution was pumped for 15 min at time point 15 min (K^+^ administration) which led to K^+^ response between time points 75 and 135 min. At time point 120 min, amphetamine (100 μM)-containing perfusion solution was pumped for 15 min (Amphet. administration) which led to amphetamine response between time points 180 and 255 min. The lag time between the administration of a stimulation solution and the corresponding response is due to slow flow rate of the perfusion solution (2 μl/min) through microdialysis tubes which have to be long in case of freely-moving animals (i.e., dead volume of the tubes is high)

Microdialysis experiments were carried out in freely-moving rats 1 and 3 weeks after the stereotaxic surgery. Before experiments, all probes (BASi MD-2200, Bioanalytical Systems, membrane length 2 mm) were tested for in vitro recovery at room temperature to ensure their proper function. However, in vivo dialysate concentrations were not corrected for in vitro recoveries because corrected concentrations do not correlate to true analyte concentrations in extracellular fluid [48]. In vitro recoveries of the probes for dopamine ranged from 7.3 to 19.1%. Before the experiments, there was a 2-h stabilization period: the probe was inserted into the guide cannula, and perfusion of the membrane was started with modified Ringer solution (147 mM NaCl, 2.7 mM KCl, 1.2 mM CaCl_2_, 1.0 mM MgCl_2_, 0.04 mM ascorbic acid) at a flow rate of 2 μl/min. After the stabilization period, dialysate samples were collected every 15 min for 270 min (Fig. 1b). The samples were analyzed right after collecting using a high-performance liquid chromatography (HPLC) coupled with an electrochemical detector as described below. Analyte concentrations in the first four samples (time points 15, 30, 45, and 60 min) were used to calculate baseline levels (as averages) for dopamine and its main metabolites 3,4-dihydroxyphenylacetic acid (DOPAC) and homovanillic acid (HVA). To stimulate dopamine release from nerve terminals, two different stimulation solutions were administrated via reverse dialysis. First, the Ringer solution was changed into 100 mM potassium solution (27.5 mM NaCl, 100 mM KCl, 1.2 mM CaCl_2_, 1.0 mM MgCl_2_, 0.04 mM ascorbic acid) at time point 15 min. This high-potassium solution was pumped for 15 min after which it was changed back to the Ringer solution. At time point 120 min, the Ringer solution was replaced with 100 μM D-amphetamine solution for 15 min. After this, the Ringer solution was used until the end of the experiment. The results were analyzed as percent changes of the analyte concentrations compared to the corresponding baseline levels. If the concentration of any analyte in the last two baseline samples (time points 45 and 60 min) differed more than 20% of the average, the rat was excluded from the data. After the first microdialysis experiment, the probe was removed from the brain. After the second experiment, rat was sacrificed and the brain was excised and frozen on dry ice. The correct placements of the microdialysis probes were verified histologically from 90-μm-thick coronal brain sections which were cut with a sliding microtome (Leica CM3050, Leica Instruments GmbH, Germany) and fixed on gelatin-chrome-alume-coated microscope slides. Data only from the rats with accurate probe placements in the dorsal striatum were included in the analyses.

### Quantification of Dopamine and Metabolites from Microdialysis Samples

The analysis of samples collected in microdialysis experiments and in in vitro recovery tests was performed with slight modifications from the methods described earlier [49, 50]. The concentrations of dopamine, DOPAC and HVA in the samples were analyzed with a HPLC system equipped with an ESA Coulochem II electrochemical detector and a model 5014B microdialysis cell (ESA Biosciences Inc., MA, USA). Dopamine was reduced with an amperometric detector (potential − 100 mV) after being oxidized with a coulometric detector (+ 300 mV); DOPAC and HVA were oxidized with the coulometric detector. Dialysate samples of 25 μl were injected into the column (Kinetex C18, 2.6 μm, 100 Å, 50 × 4.6 mm; Phenomenex Inc., CA, USA) with an autoinjector (Prominence, SIL-20 AC, Shimadzu Co., Japan). The column was protected with SecurityGuard Ultra filter (Phenomenex) and its temperature was kept at 45 °C with a column heater (Croco-Cil, Cluzeau Info Labo, France). The mobile phase was a mixture of 0.1 M NaH_2_PO_4_ buffer, pH 4.0, 100 mg/l octanesulphonic acid, approximately 5% (*v*/*v*) of methanol and 0.2 M ethylenediaminetetraacetic acid (EDTA). The flow rate of the mobile phase was kept constant at 1.0 ml/min with an isocratic pump (Jasco PU-2080 Plus, Jasco Co., Essex, UK) equipped with two pulse dampers (SSI LP-21, Scientific Systems Inc., PA, USA). The chromatograms were processed with chromatogram integration software (Azur 4.0, Datalys, France).

### In Vivo TH Activity Experiment

Seven days after an intrastriatal injection of NTFs (10 μg in 5 μl) or vehicle (5 μl), rats were administered with 3-hydroxybenzylhydrazine (NSD1015) 100 mg/kg, i.p. (Sigma-Aldrich Chemie GmbH, Germany) to inhibit aromatic amino acid dopa decarboxylase (AADC) in the brain [51]. Rats were decapitated 30 min after the NSD1015 injection; the brains were excised rapidly and rinsed with ice-cold physiological saline solution. Dorsal striatum samples were collected bilaterally from 2-mm coronal slices using an ice-cooled rat brain matrix (Stoelting) and a 3-mm sample corer (Fine Science Tools GmbH, Heildelberg, Germany) and frozen immediately on dry ice. The samples were weighed and homogenized with a sonicator (GM35-400, Rinco Ultrasonics AG, Switzerland) in 500 μl of homogenization solution consisting of six parts of 0.2 M HClO_4_ and one part of antioxidant solution containing 1.0 mM oxalic acid, 0.1 M acetic acid, and 3.0 mM L-cysteine [52]. The homogenates were centrifuged at 20,800×*g* for 35 min at 4 °C (Eppendorf Centrifuge 5810R, Eppendorf AG, Germany). After the centrifuging, 300 μl of the supernatant was moved into Vivaspin® 500 filter concentrators (10,000 MWCO PES; Sartorius Stedim Biotech GmbH, Germany) and centrifuged again at 8600×*g* for 35 min at 4 °C. Filtrates containing monoamines were analyzed with a HPLC system as described below. The amount of L-3,4-dihydroxyphenylalanine (L-DOPA) in the striatum samples was calculated as nanograms per gram (ng/g) wet weight of the sample for both hemispheres.

### Quantification of L-DOPA from Striatal Tissue Samples

The concentration of L-DOPA in striatal tissue samples was measured with a HPLC system as described earlier [53]. Samples of 100 μl were injected into the column (Kinetex XD-C18, 2.6 μm, 100 Å, 100 × 4.6 mm; Phenomenex) with an autoinjector (Prominence Auto Sampler, SIL-20 AC, Shimadzu). The column temperature was kept at 45 °C with a column heater (Croco-Cil). The mobile phase was a mixture of 0.1 M NaH_2_PO_4_ buffer, pH 3.0, 150 mg/l octanesulfonic acid 4% (*v*/*v*) of methanol. An isocratic pump (ESA Model 582 Solvent Delivery Module; ESA Biosciences) equipped with a pulse damper (SSI LP-21, Scientific Systems) provided a constant flow rate of 1.0 ml/min. The analytes were detected with an electrochemical detector (ESA CoulArray Electrode Array, ESA Biosciences), and the chromatograms were processed with an integration software (CoulArray for Windows, ESA Biosciences).

### Estimation of COMT and MAO Activities

COMT, MAO-A, and MAO-B enzyme activities were measured from striatum samples 7 days after an intrastriatal injection of GDNF (10 μg in 5 μl) or vehicle (5 μl). Rats were decapitated, and the brains were excised rapidly and rinsed with ice-cold physiological saline solution. Dorsal striatum samples were collected bilaterally from 4-mm coronal slices using an ice-cooled rat brain matrix (Stoelting) and a 3-mm sample corer (Fine Science Tools) and frozen immediately on dry ice. The samples were weighed and homogenized with a sonicator (GM35-400, Rinco Ultrasonics) in 10 mM phosphate buffer (pH 7.4) in a volume of 20× wet weight of the sample. The homogenates were centrifuged at 1000×*g* for 20 min at 4 °C (Eppendorf Centrifuge 5810R). Total protein concentration was determined using bicinchoninic acid method (Pierce™ BCA Protein Assay Kit, Thermo Fisher Scientific Inc., MA, USA).

Total COMT activity assay was performed as described earlier [54, 55]. The enzyme suspension was incubated for 30 min at 37 °C in 100 mM phosphate buffer (pH 7.4) containing 5 mM MgCl_2_, 200 μM S-adenosyl-L-methionine (Sigma Chemical Co., MO, USA), and 500 μM 3,4-dihydroxybenzoic acid (Sigma). The reaction was stopped by adding ice-cold 4 M perchloric acid. Samples were centrifuged at 5530×*g* for 10 min at 4 °C after which supernatant was filtered through 0.45-μm syringe filter (Millex®-HA, Millipore, Merck KGaA, Darmstadt, Germany) and diluted 1:20 in 0.4 M perchloric acid. The reaction products, vanillic and isovanillic acid, were analyzed with a HPLC system equipped with an ESA Coulochem II electrochemical detector (detector potential + 500 mV) and a model 5011A analytical cell (ESA Biosciences). An autoinjector (Prominence SIL-20AC, Shimadzu) was used to inject 10 μl of the samples into the column (Spherisorb® ODS2, C18, 3 μm, 4.6 × 100 mm; Waters Spherisorb, MA, USA). The column temperature was kept at 35 °C with a column heater (Croco-Cil). The mobile phase consisted of 0.1 M Na_2_HPO_4_ buffer (pH 3.3), 0.15 mM EDTA and 25% methanol. The flow rate of the mobile phase was set to 1.0 ml/min and kept constant with an isocratic pump (Jasco PU-2080 Plus, Jasco). The chromatograms were processed with an integration software (Azur 5.0, Datalys). Total COMT activity was calculated as picomoles of vanillic acid formed in 1 minute per milligram of protein in the sample.

MAO-A and MAO-B activities were determined with Monoamine Oxidase Assay Kit (MAK136, Sigma-Aldrich) according to the manufacturer’s instructions. In the assay, MAO-A and -B react with p-tyramine forming H_2_O_2_ which is determined by a fluorimetric method. To isolate MAO-A and MAO-B activities, isoform-specific inhibitors were used: MAO-A activity was assayed by adding 5 μM of MAO-A-specific inhibitor clorgyline into the control well and subtracting the remaining MAO-B activity from the total MAO activity in the sample well without inhibitors. Likewise, MAO-B activity was assayed by adding 5 μM of MAO-B-specific inhibitor pargyline into the control well and subtracting the remaining MAO-A activity from the total MAO activity. Black 96-well plates with clear bottom were used in the assay. The fluorescence of the samples and H_2_O_2_ standard curve was measured with a multi-well plate reader (Victor2, 1420 Multilabel Plate Reader, PerkinElmer Inc., MA, USA) using an excitation wavelength of 530 nm and a detection wavelength of 590 nm.

### Statistical Analysis

Data from the microdialysis experiments were analyzed with analysis of variance (ANOVA) for repeated measures followed by Ryan-Einot-Gabriel-Welsch F (REGWF) post hoc test. One-way ANOVA followed by REGWF post hoc test was used to compare the differences in the baseline concentrations between the treatment groups as well as to analyze results from the in vivo TH activity experiment. Differences in the baseline concentrations within the treatment groups 1 and 3 weeks after the surgery were analyzed with paired two-tailed Student’s *t* test. Results from the COMT and MAO activity experiments were analyzed with unpaired two-tailed Student’s *t* tests. All analyses were conducted with SPSS® Statistics 24 software (IBM SPSS Inc., IL, USA). Exclusion criterion used in the data analyses was mean ± 2 × standard deviation. Results are expressed as mean ± SEM and considered to be significant at *p* < 0.05.

## Results

### Unaltered Baseline Levels of Dopamine and Its Metabolites After NTF Treatments

The effects of intrastriatally injected NTFs on dopamine release from nigrostriatal dopaminergic neurons were examined in two consecutive microdialysis experiments, at 1 and 3 weeks after the stereotaxic surgery, in freely-moving rats. The extracellular baseline levels of dopamine, DOPAC, or HVA did not differ significantly between the treatment groups either at 1 or 3 weeks after the surgery. However, the baseline levels of dopamine and its metabolites were significantly lower in most of the treatment groups when measured at 3 weeks after the surgery as compared with the concentrations measured at 1 week after the surgery (Table 1).

**Table 1:** Dopamine, 3,4-dihydroxyphenylacetic acid (DOPAC), and homovanillic acid (HVA) concentrations (nM) in the baseline samples at 1 and 3 weeks after the surgery. Baseline value for each analyte is shown as average of the concentrations in the baseline samples (at time points 15, 30, 45, and 60 min); mean ± SEM; at week 1, *n* = 10 in each group; at week 3, *n* = 8–10 in each group

		Week 1		Week 3	
		Mean	SEM	Mean	SEM
Dopamine (nM)	VEH	1.43	0.26	0.80 *	0.16
	GDNF	1.54	0.47	0.52	0.10
	CDNF	1.05	0.18	0.65 *	0.16
	MANF	0.92	0.17	0.68	0.07
DOPAC (nM)	VEH	539.61	59.97	464.43	72.36
	GDNF	560.44	96.49	311.29 *	58.60
	CDNF	553.02	56.58	385.77 *	60.50
	MANF	620.39	47.10	443.76 *	42.34
HVA (nM)	VEH	323.93	36.59	303.21	44.11
	GDNF	475.58	88.83	238.65 **	40.58
	CDNF	351.30	43.21	252.38 *	37.90
	MANF	368.47	28.70	293.61 *	32.18

*p < 0.05, **p < 0.01 (paired two-tailed Student’s *t* test, between week 1 and week 3 baseline concentrations)

### Elevated Stimulus-Evoked Release of Dopamine in MANF-Treated Rats

**Fig. 2:**
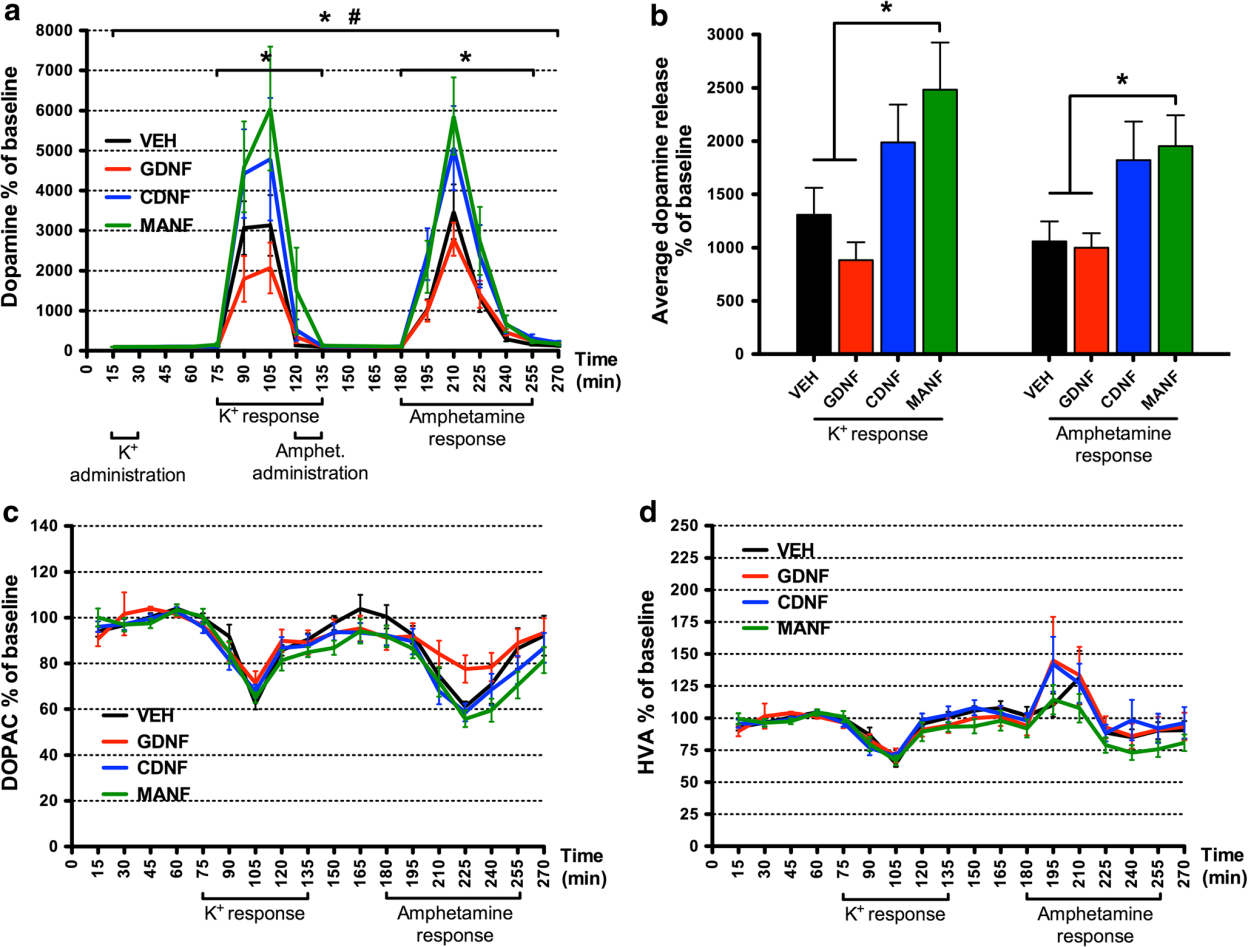
Extracellular striatal concentrations of dopamine and its main metabolites 3,4-dihydroxyphenylacetic acid (DOPAC) and homovanillic acid (HVA) measured using brain microdialysis 1 week after an intrastriatal injection of GDNF, CDNF, MANF, or vehicle. **a** In MANF-treated rats, both potassium-evoked (75–135 min) and amphetamine-evoked (180–255 min) release of dopamine was elevated when compared to vehicle- and GDNF-treated rats. In addition, the overall (15–270 min) extracellular dopamine concentration was elevated in MANF group compared to vehicle and GDNF groups, and in CDNF group compared to GDNF group (*p < 0.05 MANF vs. VEH and MANF vs. GDNF; #*p* < 0.05 CDNF vs. GDNF; REGWF post hoc analysis after ANOVA for repeated measures). **b** Average dopamine overflow during potassium and amphetamine responses was augmented in MANF-treated rats when compared to vehicle- and GDNF-treated rats (*p < 0.05 MANF vs. VEH and MANF vs. GDNF; REGWF post hoc analysis after one-way ANOVA). **c**, **d** NTFs did not have significant effects on extracellular DOPAC or HVA concentration at 1 week after the injection. The period when high-potassium and amphetamine perfusion solutions were pumped (K^+^ and Amphet. administration) (**a**) and the period of potassium and amphetamine responses (**a**, **c**, **d**) are depicted under the *x*-axes. Results are shown as % of baseline value (= 100%); mean ± SEM; *n* = 10 in each group

To study the ability of NTFs to alter stimulus-evoked release of dopamine in the striatum, dopaminergic nerve terminals were first depolarized by administrating high concentration of potassium via reverse dialysis which caused an extensive increase in the extracellular concentration of dopamine in all treatment groups (Fig. 2a, b). After a recovery period, administration of amphetamine through the microdialysis probe drained dopaminergic vesicles and reversed the function of dopamine transporter (DAT) in the nerve terminals [56], thus inducing another notable increase in dopamine release.

One week after the surgery, potassium- and amphetamine-evoked release of dopamine was significantly elevated in MANF-treated rats as compared with the vehicle- and GDNF-treated animals (ANOVA for repeated measures 75–135 min: *F*_3,36_ = 4.874; *p* = 0.006; REGWF *p* < 0.05; 180–255 min: *F*_3,36_ = 3.683; *p* = 0.021; REGWF *p* < 0.05) (Fig. 2a). In addition, extracellular dopamine concentration significantly differed between the treatment groups during the whole experiment (ANOVA for repeated measures 15–270 min: *F*_3,36_ = 4.678; *p* = 0.007). According to REGWF’s post hoc test, the differences were between MANF- and vehicle-, MANF- and GDNF-, and CDNF- and GDNF-treated animals (*p* < 0.05). We also compared total potassium and amphetamine responses between the treatment groups by analyzing average increase in dopamine release during the stimulus responses (Fig. 2b). The results from these analyses supported our findings: Average potassium-evoked dopamine overflow was augmented in MANF-treated rats as compared with the vehicle- and GDNF-treated rats (one-way ANOVA *F*_3,36_ = 4.874; *p* = 0.006; REGWF *p* < 0.05). Congruently, average amphetamine-evoked dopamine overflow was augmented in MANF group when compared to the vehicle and GDNF groups (one-way ANOVA *F*_3,36_ = 3.683; *p* = 0.021; REGWF *p* < 0.05).

Three weeks after the surgery, we did not find statistically significant differences between the treatment groups. Figures showing microdialysis results at 3 weeks after the surgery are presented in supplementary material (Fig. 5).

DOPAC concentration in the extracellular fluid decreased during the potassium and amphetamine responses compared to the baseline level in all treatment groups as reported earlier [57, 58] (Fig. 2c, supplementary material Fig. 5b). Similarly, also HVA concentration decreased during the potassium response but increased during the amphetamine response (Fig. 2d, supplementary material Fig. 5c). No statistical differences, however, were observed between the treatment groups in extracellular concentrations of DOPAC or HVA.

### Increased Dopamine Turnover in MANF-Treated Rats

**Fig. 3:**
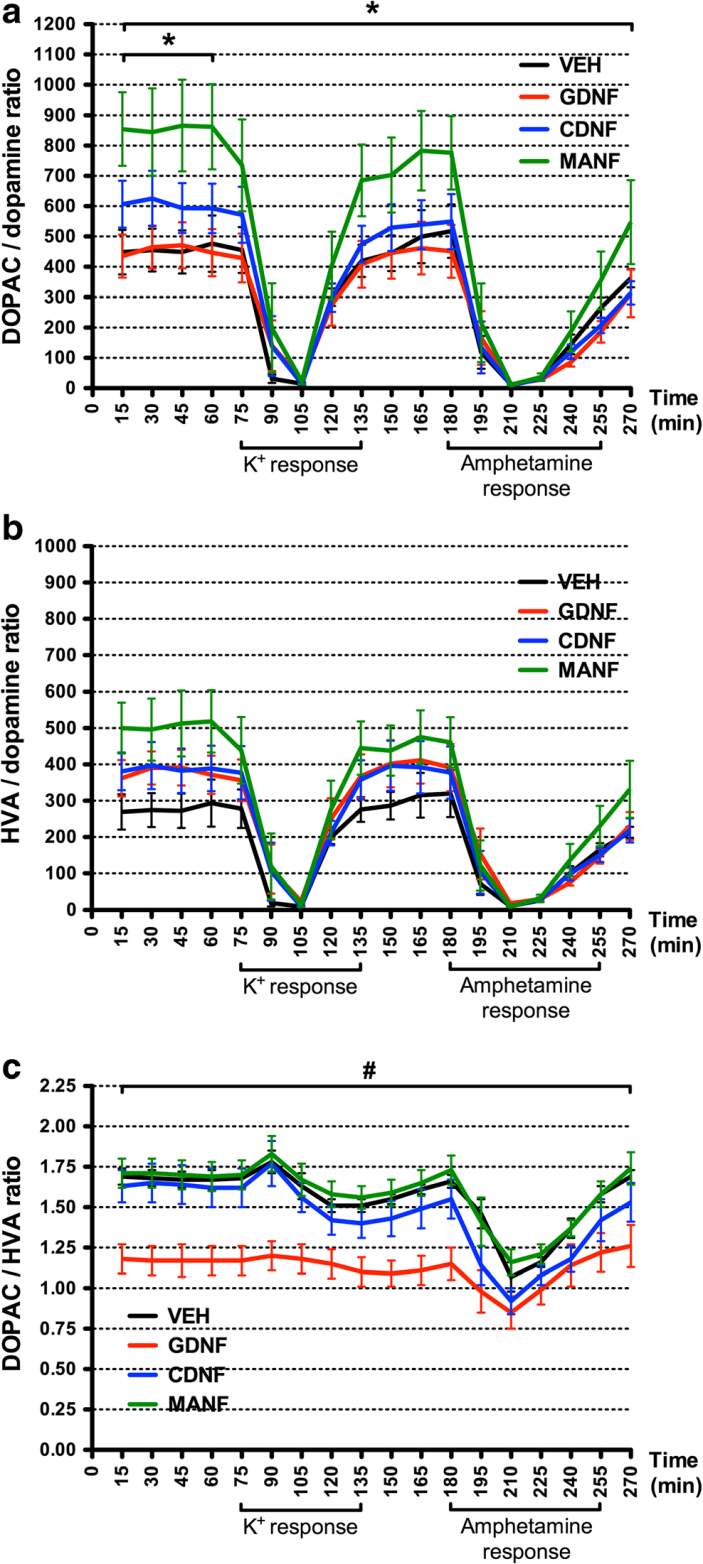
Dopamine turnover analyzed 1 week after an intrastriatal injection of GDNF, CDNF, MANF, or vehicle. DOPAC/dopamine, HVA/dopamine, and DOPAC/HVA ratios were calculated as ratios of the analyte concentrations in the microdialysis samples. **a** DOPAC/dopamine turnover was enhanced in MANF-treated rats as compared to the rats treated with vehicle or GDNF during the whole experiment (15–270 min) and during the baseline (15–60 min) (*p < 0.05 MANF vs. VEH and MANF vs. GDNF; REGWF post hoc analysis after ANOVA for repeated measures). **b** There were no significant changes in HVA/dopamine turnover. **c** Less DOPAC was formed compared to HVA in GDNF-treated rats as measured by DOPAC/HVA ratio which was significantly smaller in GDNF group than in the other treatment groups during the whole experiment (15–270 min) (#*p* < 0.05 GDNF vs. VEH, GDNF vs. CDNF and GDNF vs. MANF; REGWF post hoc analysis after ANOVA for repeated measures). The period of potassium and amphetamine responses are depicted under the *x*-axes. Results are shown as concentration ratios; mean ± SEM; *n* = 10 in each group

To study if NTFs were able to alter dopamine metabolism in the striatum, we determined dopamine turnover by calculating DOPAC/dopamine and HVA/dopamine ratios from the microdialysis samples. One week after the surgery, MANF produced marked increase in dopamine turnover as evaluated by DOPAC/dopamine ratio (Fig. 3a). The ratio was significantly higher in MANF-treated rats as compared with the vehicle- and GDNF-treated rats during the whole experiment (ANOVA for repeated measures 15–270 min: *F*_3,36_ = 3.065; *p* = 0.040; REGWF *p* < 0.05) as well as in the baseline samples (ANOVA for repeated measures 15–60 min: *F*_3,36_ = 3.868; *p* = 0.017; REGWF *p* < 0.05). Three weeks after the surgery, there were no differences between the treatment groups in DOPAC/dopamine ratio (supplementary material Fig. 6a). We did not see any significant differences in HVA/dopamine ratio between the treatment groups either 1 or 3 weeks after the surgery (Fig. 3b, supplementary material Fig. 6b).

We also calculated the ratio of the metabolites from the microdialysis samples. Unexpectedly, 1 week after the surgery, DOPAC/HVA ratio was significantly reduced in GDNF-treated rats compared to all the other treatment groups during the whole experiment (ANOVA for repeated measures 15–270 min: *F*_3,36_ = 7.397; *p* = 0.001; REGWF *p* < 0.05) (Fig. 3c). Three weeks after the surgery, DOPAC/HVA ratio was still smaller in GDNF group than in the other groups but the difference did not reach significance (ANOVA for repeated measures 15–270 min: *F*_3,32_ = 2.537; *p* = 0.074) (supplementary material Fig. 6c). The changed DOPAC/HVA ratio suggests that GDNF injection alters the activity of dopamine metabolizing enzymes COMT and MAO.

### Increased In Vivo TH Activity in Rats Treated with GDNF

As we found elevated stimulus-evoked release of dopamine in MANF-, but not in GDNF-, treated rats, we wanted to investigate the effect of the NTF injection on in vivo activity of TH. To that end, we inhibited AADC enzyme in the brain with a blood-brain barrier passing inhibitor NSD1015 1 week after the NTF injection. Half an hour after AADC inhibition, we collected tissue samples from the dorsal striatum and quantified accumulated L-DOPA in the samples. In this experiment, the amount of L-DOPA accumulated into the striatum provides a direct measure for L-DOPA production rate, which reflects the in vivo activity of phosphorylated and non-phosphorylated TH in the nigrostriatal pathway [51].

**Fig. 4:**
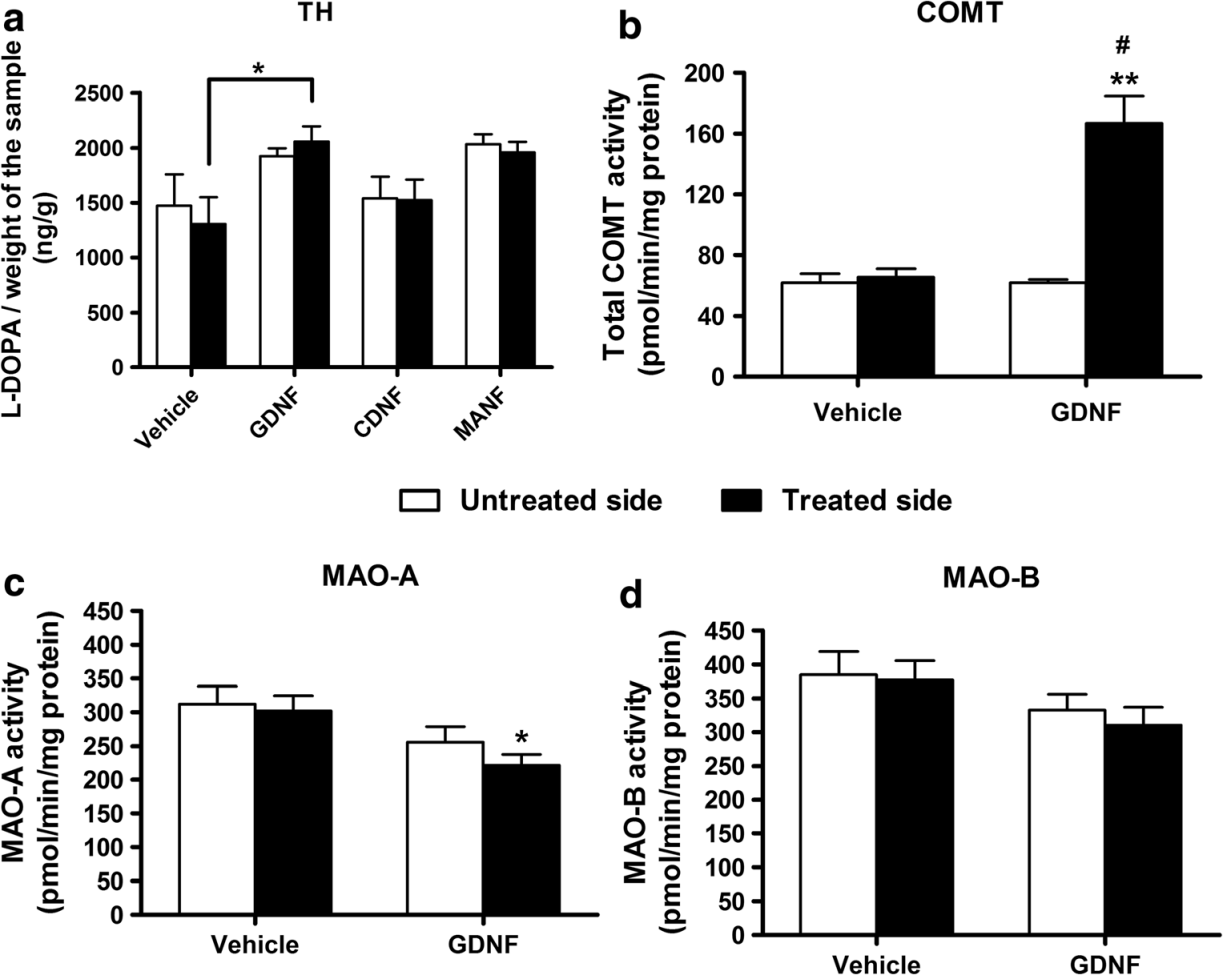
Effect of NTF treatment on the activity of dopamine neurochemistry-regulating enzymes. **a** In vivo TH activity was assayed 1 week after an intrastriatal injection of GDNF, CDNF, MANF, or vehicle by collecting striatal tissue samples 30 min after the inhibition of aromatic amino acid dopa decarboxylase with NSD1015 (100 mg/kg, i.p.) and analyzing the amount of accumulated L-DOPA in the samples. L-DOPA was synthetized more in rats treated with GDNF as compared to the vehicle-treated rats which indicates increased TH activity in GDNF-injected striata (*p < 0.05 GDNF vs. VEH; one-way ANOVA followed by REGWF post hoc test). **b**–**d** Due to significantly reduced DOPAC/HVA ratio in GDNF-treated rats seen in the microdialysis experiments, the effect of GDNF on COMT, MAO-A, and MAO-B activity was determined from striatal tissue samples collected 1 week after an intrastriatal injection of GDNF or vehicle. **b** GDNF significantly increased total COMT activity in the treated striatum as compared to the vehicle-treated rats (**p < 0.001; unpaired two-tailed Student’s *t* test) as well as compared to the untreated side (#*p* < 0.001; paired two-tailed Student’s *t* test). **c** GDNF significantly reduced MAO-A activity in the treated striatum as compared to the vehicle-treated rats (* *p* = 0.011; unpaired two-tailed Student’s *t* test). **d** MAO-B activity was also reduced in rats treated with GDNF but not significantly. Results are shown as mean ± SEM; in TH activity experiment, *n* = 6–7 in each group; in COMT and MAO activity experiments, *n* = 7–8

GDNF was able to increase TH activity as the amount of L-DOPA in the treated striatum was increased approximately by 60% as compared with the vehicle (one-way ANOVA *F*_3,22_ = 3.780; *p* = 0.025; REGWF *p* < 0.05) (Fig. 4a). MANF also tended to enhance the accumulation of L-DOPA (approximately by 50%) while CDNF had no effect. The amount of L-DOPA in treated versus untreated striatum did not differ within the treatment groups when compared with paired two-tailed Student’s *t* test.

### Increased COMT Activity and Reduced MAO Activity in Rats Treated with GDNF

Because of the significantly reduced DOPAC/HVA ratio in GDNF-treated rats, we decided to assess the effect of GDNF on dopamine-metabolizing enzymes COMT, MAO-A, and MAO-B. To study this, GDNF or vehicle was unilaterally injected into the dorsal striatum and 1 week later, striatal tissue samples were collected for ex vivo enzyme activity assays.

GDNF increased the total activity of COMT by 155% in the treated striatum when compared to the vehicle-treated rats (unpaired two-tailed Student’s *t* test *t*(13) = − 5.159; *p* < 0.001) and by 170% when compared to the untreated striatum (paired two-tailed Student’s *t* test *t*(7) = 6.041; *p* < 0.001) (Fig. 4b). GDNF also reduced MAO-A activity in the treated striatum by 27% when compared to the vehicle-treated controls (unpaired two-tailed Student’s *t* test *t*(14) = 2.944; *p* = 0.011) (Fig. 4c). GDNF had a modest reducing effect on MAO-B activity (approximately 18% as compared to the vehicle-treated controls), but the difference remained insignificant (unpaired two-tailed Student’s *t* test *t*(14) = 1.689; *p* = 0.113) (Fig. 4d).

## Discussion

The main findings in this study were as follows: (i) Intrastriatal injection of MANF elevated stimulus-evoked release of dopamine in the striatum 1 week after the injection. Elevated dopamine release was accompanied by enhanced DOPAC/dopamine turnover in MANF-treated rats. (ii) In GDNF-treated rats, stimulus-evoked release of dopamine was not changed although striatal TH activity was increased. At the same time, DOPAC/HVA ratio was decreased apparently due to increased COMT activity and decreased MAO-A activity.

To be able to assess different presynaptic release mechanisms, we utilized a microdialysis protocol with two distinct stimuli (high concentration of K^+^ and amphetamine) to evoke dopamine release from nigrostriatal nerve endings. We saw smaller differences in dopamine overflow between the treatment groups during the amphetamine response than during the potassium response (Fig. 2a). High concentration of potassium depolarizes nerve terminals and causes fusion of vesicles close to the presynaptic membrane in a calcium-dependent manner [59]. This pool of presynaptic dopamine is considered to be readily releasable. Amphetamine, on the contrary, is known to deplete vesicular stores of dopamine and reverse the function of DAT causing calcium independent release of dopamine [56, 59]. Thus, amphetamine stimulus gives an estimate of the total amount of dopamine stored in nerve terminals. Our results may indicate that MANF can enhance the dynamics of calcium mediated membrane fusion of presynaptic dopamine vesicles or increase the proportion of readily releasable vesicles as demonstrated by elevated potassium-evoked release of dopamine.

One week after the NTF injection, stimulus-evoked release of dopamine was elevated in MANF-treated rats suggesting increased sprouting of dopaminergic fibers (Fig. 2a). This may not be the case, however, since Voutilainen et al. showed no effect of 2-week intrastriatal infusion of MANF on TH-immunoreactivity in the substantia nigra or striatum in intact rats [15]. Thus, it is more likely that MANF enhances dopaminergic neurotransmission through presynaptic storage or release mechanisms rather than through sprouting. For example, GDNF has been proposed to facilitate synaptic transmission by modulating the quantal size of neurotransmitter release [19], potentiating Ca^2+^ influx [22, 60], and inhibiting A-type K^+^-channels [21], thereby potentiating excitability of neurons. It is possible that MANF has same type of modulatory effects on the function of nerve terminals although its possible effects on ion channels still remain to be clarified.

Contrary to the previous studies, the stimulus-evoked release of dopamine was not increased in GDNF-treated animals [24–26, 28, 29]. The previous studies were conducted under general anesthesia. Anesthetics are known to have marked effects on neurotransmission which can explain the differing results from the present study [30, 31]. Similarly to our results, by using freely-moving rats with only a brief metofane anesthesia at the beginning of microdialysis experiment, Xu and Dluzen did not see significant differences between GDNF- and vehicle-injected rats [61]. In a microdialysis study with freely-moving mice, potassium-evoked release of dopamine did not differ between wild-type and MEN2B mice that have constantly active RET [62]. These observations are in line with our results. DAT activity has been shown to be markedly increased in MEN2B mice and in GDNF hypermorphic mice overexpressing GDNF [62, 63]. Therefore, it can be hypothesized that in the present study, increased DAT activity after GDNF treatment results in enhanced clearance of extracellular dopamine after potassium-stimulus and thus nullifies the dopaminergic transmission enhancing effect of GDNF. In addition, during amphetamine response, when dopamine reuptake through DAT and metabolism through MAO are inhibited, the role of COMT in dopamine turnover is pronounced. As GDNF was shown to increase COMT activity, it can be speculated that amphetamine-evoked dopamine release was diminished in GDNF-treated rats due to increased metabolism through COMT.

Baseline concentration of dopamine and its metabolites remained unchanged between the treatment groups at 1 and 3 weeks after the NTF injection (Table 1). This result is in line with earlier microdialysis experiments: there were no differences in the basal extracellular dopamine concentration between GDNF- and vehicle-treated rats [24, 61] or between MEN2B and wild-type mice [62]. The unchanged baseline concentrations may result from effective homeostatic mechanisms after NTF treatments, including enhanced uptake of dopamine through DAT. It is also possible that dopamine is stored more in terminal vesicles in NTF-treated animals, while the tonic release of dopamine remains unchanged during the baseline.

To study if the elevated release of dopamine in MANF-injected rats was due to enhanced synthesis of dopamine, we determined the effect of the NTFs on in vivo TH activity. Because of the fact that unilaterally injected NTFs have bilateral effects [42, 64, 65], we compared the amount of L-DOPA only in the treated striata between the treatment groups. We saw significantly increased TH activity in GDNF-treated rats, measured as accumulated striatal L-DOPA following inhibition of AADC [51] (Fig. 4a). MANF also seemed to have an increasing effect on TH activity, but this effect was not significant. Thus, TH activity cannot solely explain the significant elevation in the stimulus-evoked dopamine release seen in MANF-treated rats. The effect of GDNF on TH activity, on the other hand, is in line with an earlier observation according to which continuous RET activation in MEN2B mice increases in vivo TH activity [62]. Although exogenous GDNF has been shown to downregulate the total expression of TH in dopamine neurons, it can also increase the phosphorylation of TH and consequently the activity of the enzyme [28, 42–46]. Thus, the differences in TH activity between the treatment groups can be due to different ability of NTFs to enhance the phosphorylation of TH. The downregulation of TH after GDNF administration may be a compensatory response to its increased phosphorylation and activity. Increased TH activity in GDNF-treated rats may also be due to decreased amount of dopamine in nerve terminals which might affect the activity of TH through feed-back mechanisms as speculated by Georgievska et al. [44].

DOPAC/dopamine ratio was measured from the microdialysis samples as an indicator of dopamine metabolism. The turnover of dopamine into DOPAC was significantly increased in MANF group as compared to vehicle and GDNF groups 1 week after the NTF injection (Fig. 3a). Moreover, HVA/dopamine turnover seemed to be increased in MANF group but the differences were not statistically significant (Fig. 3b). Increased DOPAC/dopamine ratio can be considered a sign of enhanced dopaminergic neurotransmission [67, 68]. In earlier studies, DOPAC/dopamine and HVA/dopamine ratios in striatal and nigral tissue samples were increased in GDNF-treated animals [66–69], but Hebert et al. reported unchanged dopamine turnover in GDNF-treated rats [24]. Apart from enhanced dopaminergic neurotransmission, the increased DOPAC/dopamine turnover after MANF treatment can be a consequence of augmented tonic release of dopamine outside the stimulus responses. As the metabolism of dopamine is efficient, increased amount of extracellular dopamine might lead to the higher turnover ratio.

Interestingly, we saw a significantly reduced DOPAC/HVA ratio in GDNF-treated rats at 1 week after the NTF injection (Fig. 3c). To further elucidate this phenomenon, we tested the possible effect of GDNF on the activity of the dopamine-metabolizing enzymes COMT, MAO-A, and MAO-B. GDNF significantly increased total COMT activity and decreased MAO-A activity in the striatum when compared to the vehicle, giving a logical explanation for the reduced DOPAC/HVA ratio (Fig. 4b, c). Helkamaa et al. have demonstrated that LPS-induced microglia activation results in increased COMT activity in the rat brain [70]. In the present study, however, microglia activation due to surgical procedures cannot explain the increased activity of COMT in GDNF-injected rats, because the vehicle injection did not cause any changes in total COMT activity when compared to the non-injected side (Fig. 4b). Consequently, GDNF seems to have a direct increasing effect on COMT activity or expression or both.

One possible factor behind the divergent effects of the NTFs on dopamine neurochemistry seen in our experiments can be their different diffusion properties within brain parenchyma. Volume of distribution of GDNF in the brain is limited by its high affinity binding to heparan sulfate proteoglycans in the extracellular matrix and cell surfaces [71]. CDNF and especially MANF, on the other hand, have shown to have better diffusion properties in the rat brain when compared to GDNF [14, 15]. Thus, efficient distribution of MANF in the striatum may explain its more pronounced effect on dopamine release. Another explanation for the divergent effects can be different production methods of the NTFs. GDNF was produced in *E. coli* bacterial cells, CDNF in Sf9 insect cells and MANF in CHO mammalian cells. Proteins produced in bacterial cells are not glycosylated after the translation whereas proteins produced in insect or mammalian cells can be glycosylated. Glycosylation may affect the activity and diffusion properties of the proteins in the brain. However, non-glycosylated GDNF produced in *E. coli* has been shown to have full biological activity [8]. According to our mass spectrometer analysis neither CDNF nor MANF used here were glycosylated making them comparable with the GDNF of bacterial origin. Nevertheless, it can be speculated that recombinant NTFs produced in mammalian cells may have stronger biological activity than NTFs produced in other cell lines [72].

MANF and CDNF have been proposed to work under the condition of ER stress or inflammation [38–41]. The microdialysis protocol used here indeed causes ER stress and inflammation due to mechanical damage around the sampling site. The damage also results in local degeneration of nerve terminals, edema, changes in bloodstream, and gliosis around the probe membrane [73]. Repeated insertion and removal of the probe may affect the results of the second microdialysis due to pathological changes around the perfusion area; glial scar forms a diffusion barrier for the analytes which may explain the general decline in the baseline analyte concentrations 3 versus 1 week after the NTF injection (Table 1). The activation of MANF and CDNF in ER stress conditions might be a part of the explanation why they had different effects than GDNF in this study setting. In addition, the mechanical damage caused by the implantation of the guide cannula right after the NTF injection causes an inevitable disruption of the blood-brain barrier. Due to this disruption, antibodies neutralizing exogenous NTFs may invade into the brain abolishing part of the effect of the NTFs and causing unexpected variation to the results.

To this day, the cornerstone of the treatment of PD has been pharmacological substitution of striatal dopamine with initially good efficacy, but no effect on disease progression. NTFs are regarded as the first potential disease modifying therapy for PD as they are able to halt the progression of neurodegeneration and restore aberrant neuronal function in various experimental settings. However, clinical trials with NTFs show conflicting results. Therefore, it is important to better understand the effects of NTFs on dopaminergic functions of non-lesioned brain. Our current results reveal divergent biological effects of exogenously administrated GDNF, CDNF, and MANF. MANF is able to potentiate stimulus-evoked dopaminergic neurotransmission and enhance dopamine turnover in the brain of freely-moving rats. GDNF, on the other hand, increases the activity of TH and COMT and decreases the activity of MAO-A. This study gives an insight into the long-lasting changes in dopamine synthesis, release and metabolism after a single intrastriatal NTF injection which is highly relevant information for the development of novel therapeutic strategies for neurodegenerative diseases. However, further studies are needed to clarify the cellular mechanisms by which the NTFs produce their effects on neuronal homeostasis seen in this study.

## Electronic supplementary material

Supplementary Figure 5 Extracellular striatal concentrations of dopamine and its main metabolites 3,4-dihydroxyphenylacetic acid (DOPAC) and homovanillic acid (HVA) measured using brain microdialysis three weeks after an intrastriatal injection of GDNF, CDNF, MANF or vehicle. (**a**) During the second microdialysis experiment at three weeks after the surgery there were no more statistically significant differences between the treatment groups in stimulus-evoked release of dopamine although potassium response (75-135 min) still tended to be augmented in MANF-treated rats (p = 0.084; ANOVA for repeated measures). (**b**, **c**) NTFs did not have significant effects on extracellular DOPAC or HVA concentration at three weeks after the injection. The period when high-potassium and amphetamine perfusion solutions were pumped (K+ and Amphet. administration) (**a**) and the period of potassium and amphetamine responses (**a**-**c**) are depicted under the x-axes. Results are shown as % of baseline value (=100%); mean ± SEM; n = 8-10 in each group (GIF 74 kb)

High resolution image (TIFF 44587 kb)

Supplementary Figure 6 Dopamine turnover analyzed three weeks after an intrastriatal injection of GDNF, CDNF, MANF or vehicle. DOPAC/dopamine, HVA/dopamine and DOPAC/HVA ratios were calculated as ratios of the analyte concentrations in the microdialysis samples. (**a**) There were no more significant differences between the treatment groups in DOPAC/dopamine turnover at three weeks after the surgery. (**b**) There were no significant changes in HVA/dopamine turnover either. (**c**) Three weeks after the surgery DOPAC/HVA ratio was still reduced in rats treated with GDNF although the difference was not statistically significant anymore (p = 0.074; ANOVA for repeated measures). The period of potassium and amphetamine responses are depicted under the x-axes. Results are shown as concentration ratios; mean ± SEM; n = 8-10 in each group (GIF 105 kb)

High resolution image (TIFF 55745 kb)
