# Supplementary figures and images for: Mesencephalic Astrocyte-Derived Neurotrophic Factor (MANF) Elevates Stimulus-Evoked Release of Dopamine in Freely-Moving Rats
Source: Mol Neurobiol. 2018 Jan 18;55(8):6755–68. doi: 10.1007/s12035-018-0872-8 (PMC6061195; doi:10.1007/s12035-018-0872-8)

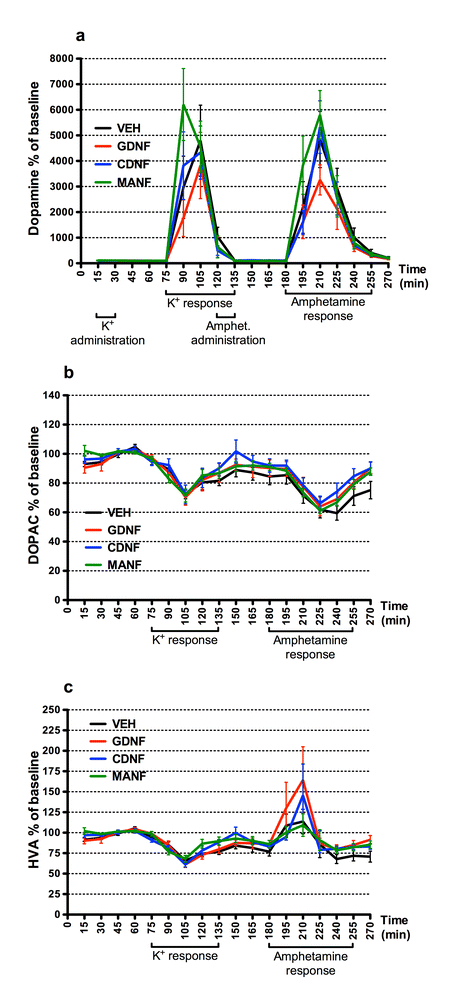

Supplement: Supplementary file 1 — Extracellular striatal concentrations of dopamine and its main metabolites 3,4-dihydroxyphenylacetic acid (DOPAC) and homovanillic acid (HVA) measured using brain microdialysis three weeks after an intrastriatal injection of GDNF, CDNF, MANF or vehicle. (a) During the second microdialysis experiment at three weeks after the surgery there were no more statistically significant differences between the treatment groups in stimulus-evoked release of dopamine although potassium response (75-135 min) still tended to be augmented in MANF-treated rats (p = 0.084; ANOVA for repeated measures). (b, c) NTFs did not have significant effects on extracellular DOPAC or HVA concentration at three weeks after the injection. The period when high-potassium and amphetamine perfusion solutions were pumped (K+ and Amphet. administration) (a) and the period of potassium and amphetamine responses (a-c) are depicted under the x-axes. Results are shown as % of baseline value (=100%); mean ± SEM; n = 8-10 in each group (GIF 74 kb) [file 12035_2018_872_Fig5_ESM.gif]

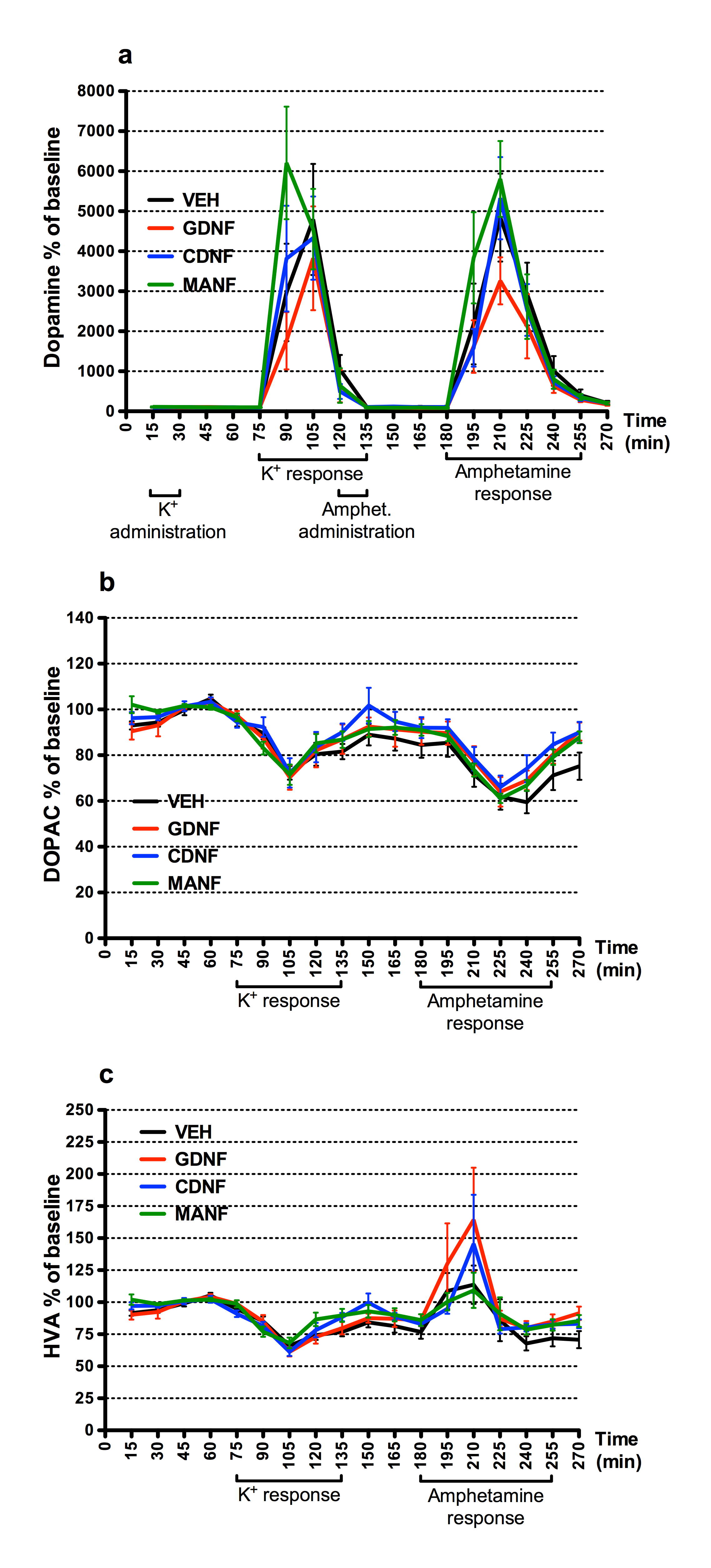

Supplement: Supplementary file 2 — High resolution image (TIFF 44587 kb) [file 12035_2018_872_MOESM1_ESM.tiff]

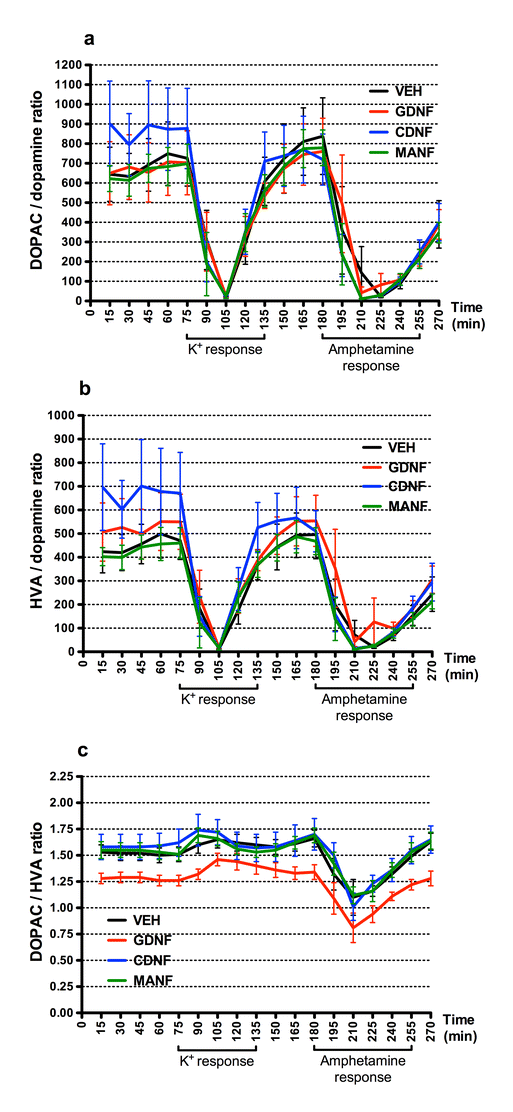

Supplement: Supplementary file 3 — Dopamine turnover analyzed three weeks after an intrastriatal injection of GDNF, CDNF, MANF or vehicle. DOPAC/dopamine, HVA/dopamine and DOPAC/HVA ratios were calculated as ratios of the analyte concentrations in the microdialysis samples. (a) There were no more significant differences between the treatment groups in DOPAC/dopamine turnover at three weeks after the surgery. (b) There were no significant changes in HVA/dopamine turnover either. (c) Three weeks after the surgery DOPAC/HVA ratio was still reduced in rats treated with GDNF although the difference was not statistically significant anymore (p = 0.074; ANOVA for repeated measures). The period of potassium and amphetamine responses are depicted under the x-axes. Results are shown as concentration ratios; mean ± SEM; n = 8-10 in each group (GIF 105 kb) [file 12035_2018_872_Fig6_ESM.gif]

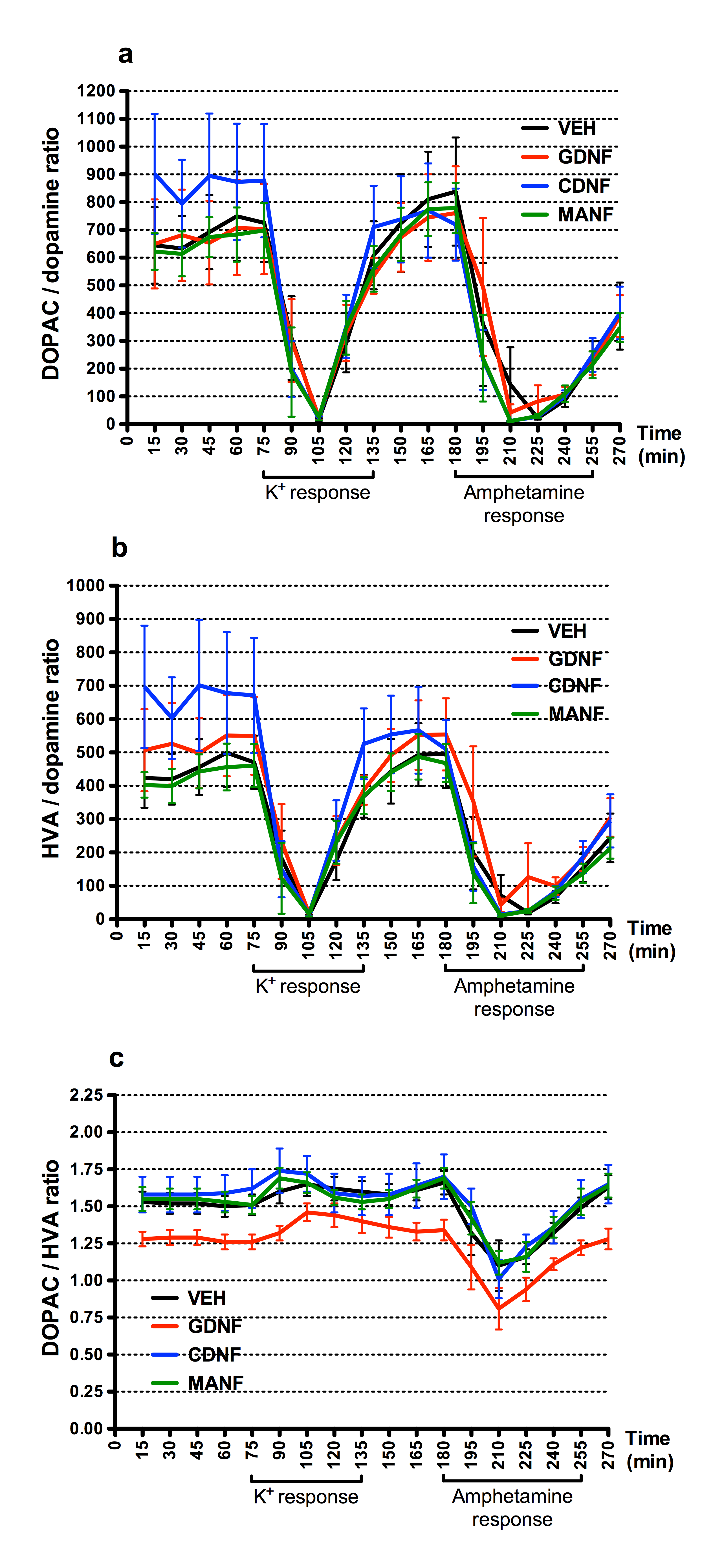

Supplement: Supplementary file 4 — High resolution image (TIFF 55745 kb) [file 12035_2018_872_MOESM2_ESM.tiff]
